# Spontaneous Abdominal Wall Hematoma Following Violent Cough: A Rare but Severe Condition—Surgical Challenges and Outcomes Regarding Three Cases and a Literature Review

**DOI:** 10.3390/reports8020049

**Published:** 2025-04-14

**Authors:** Claudiu Octavian Ungureanu, Floris Cristian Stănculea, Alexandru Iordache, Cosmin Burleanu, Valentin Titus Grigorean, Octav Ginghina, Mircea Lițescu

**Affiliations:** 1 Faculty of Dental Medicine, “Carol Davila” University of Medicine and Pharmacy, 37 Dionisie Lupu Street, 020021 Bucharest, Romania; claude.hack@gmail.com (C.O.U.); valentin.grigorean@umfcd.ro (V.T.G.); octav.ginghina@umfcd.ro (O.G.); mircea.litescu@umfcd.ro (M.L.); 2 General Surgery Department, “Sf. Ioan” Clinical Emergency Hospital, 13 Vitan-Bârzeşti Road, 042122 Bucharest, Romania; 3 “Professor Doctor Theodor Burghele” Clinical Hospital, 20 Panduri Road, 050659 Bucharest, Romania; 4 General Surgery Department, “Bagdasar-Arseni” Clinical Emergency Hospital, 12 Berceni Road, 041915 Bucharest, Romania; cosmin.burleanu@drd.umfcd.ro; 5 Department of Surgery 3, “Prof. Dr. Al. Trestioreanu” Institute of Oncology Bucharest, 022328 Bucharest, Romania

**Keywords:** hematoma, COVID-19, abdominal wall hematoma, computed tomography, cough, ultrasonography, surgery

## Abstract

**Background and Clinical Significance:** Spontaneous abdominal wall hematoma following violent coughing is a rare condition that poses a sometimes difficult therapeutic challenge, with surgical intervention often necessary in severe cases. This report aims to shed light on this rare but severe affection and raise the medical community’s interest by detailing the pathophysiology, management strategies, and outcome of spontaneous abdominal wall hematoma. **Case Presentation:** The basis for our paper was a comprehensive retrospective analysis of three cases treated in our surgical departments. We rigorously reviewed their clinical notes and imaging examinations to ensure the accuracy and reliability of our findings. **Conclusions:** These case reports underscore the challenging nature of such hematomas. Clinicians should be aware of this pathology, as it can be life-threatening. Our successful management of these cases shows that it is possible to effectively manage difficult clinical situations with timely intervention.

## 1. Introduction and Clinical Significance

Abdominal wall hematoma is an uncommon cause of abdominal pain, and it usually develops in the rectus abdominis sheath. Rectus sheath hematoma (RSH) occurs more frequently in women than in men because of reduced muscle mass compared to males. The precise frequency of RSH remains unclear, as this condition is often misdiagnosed or not diagnosed at all, especially in mild cases. Certain studies indicate a 1.5% to 2% occurrence rate among hospitalized individuals [1,2]. Its frequency has increased with the introduction of anticoagulant therapy. The rectus abdominis muscle is supplied by the epigastric arteries: one superior, arising from the internal thoracic artery, and one inferior, originating from the external iliac artery [3].

RSH results from hemorrhage caused by rupture of the epigastric arteries or their branches, or tear of the rectus abdominis muscle [4]. The clinical presentation and a positive computed tomography (CT) scan are the keys to diagnosis; often, hemoglobin shows low values. The diagnosis can be sometimes misleading, and we state that it should be included in the differential diagnosis of acute abdomen, particularly in patients presenting with blood loss. Nonoperative management (NOM) is the first-line treatment; this includes compression, rest, ice, and analgesia, and transfusion of blood in patients with anemia. If these measures fail, the next step is arterial embolization, which can control hemorrhage in most of the cases [5]. Surgery is designated in cases where endovascular treatment fails, is unavailable, or in patients with hemodynamic instability. Prognosis is good, and most of the patients recover without sequelae [6]. We present some cases of RSH that developed after a violent cough episode: one patient with no comorbidities and the others receiving anticoagulant therapy. In two cases, the conservative treatment initially recommended for every patient failed, and the medical team opted for surgery. The postoperative course of our patients was uneventful. Therefore, we stress the need for early diagnosis and timely RSH care. In addition, the outcome of this condition can be improved by being managed by a healthcare team that includes a radiologist, surgeon, physician, and assistant.

## 2. Case Presentation

**Figure 1:**
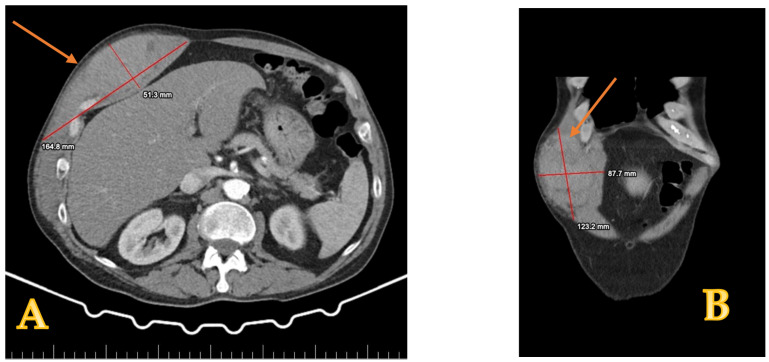
CT scan showing an abdominal mass with an elliptical shape, located in the right rectus sheath, being 123 × 87 mm in size (orange arrow). The images suggest an organized hematoma that is confined to the abdominal wall. The left image (**A**) depicts the axial aspect while the right (**B**) image presents the sagittal aspect of the CT scan.

**Case report number one:** A 65-year-old male with no prior surgeries or known comorbidities presented to the emergency department. He reported no history of trauma or anticoagulant use. For the past ten or twelve days, he had been experiencing worsening abdominal pain in his right flank. This discomfort began following a severe coughing episode two weeks earlier, coughing that progressively subsided. Clinical examination revealed a mass in the right abdomen. An ultrasound and CT scan indicated significant fluid accumulation (elliptical shape on sagittal section and ovoid on coronal section), suggesting a possible hematoma, in the right rectus abdominis muscle (Figure 1).

**Figure 2:**
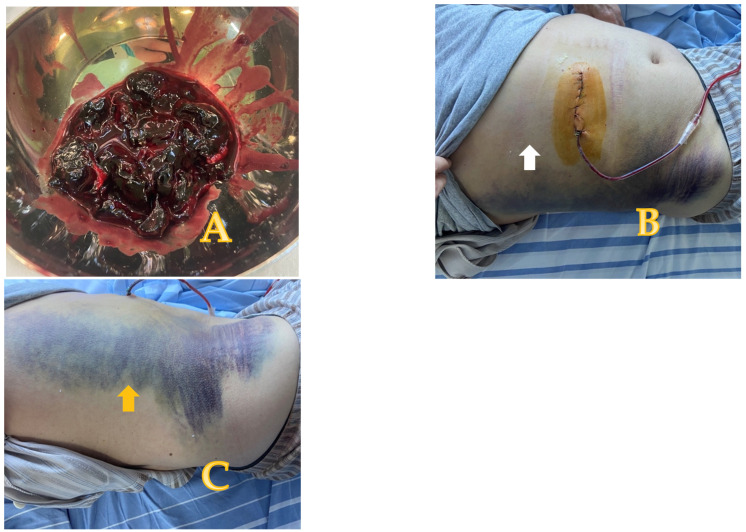
(**A**–**C**) Blood clots evacuated during surgery (**A**) and postoperative aspect: the incision along with the drain in place—white arrow (**B**). The ecchymosis in the right flank is also noticeable—yellow arrow pointing to this aspect (Grey Turner’s sign) (**C**).

The NOM initiated on admission did not alleviate the pain. We decided to manage the case using a surgical approach even though no blood transfusion was required. During surgical exploration, we discovered a sizable hematoma in the right rectus abdominis sheath, which we drained throughout the process. A bleeding branch of the inferior epigastric artery was the source of the bleeding, and the ligature of this branch attained hemostasis. A drain inserted into the remaining cavity ended the procedure and was kept until the drainage volume reached less than 50 mL. The patient was discharged from the hospital three days later (Figure 2).

During the postoperative course, a right flank ecchymosis (Grey Turner’s sign, indicating retroperitoneal bleeding) developed. The drain was kept on site for ten days and then removed. At the two-month follow-up, the patient presented no collection in the rectus abdominis sheath during the ultrasound examination.

**Figure 3:**
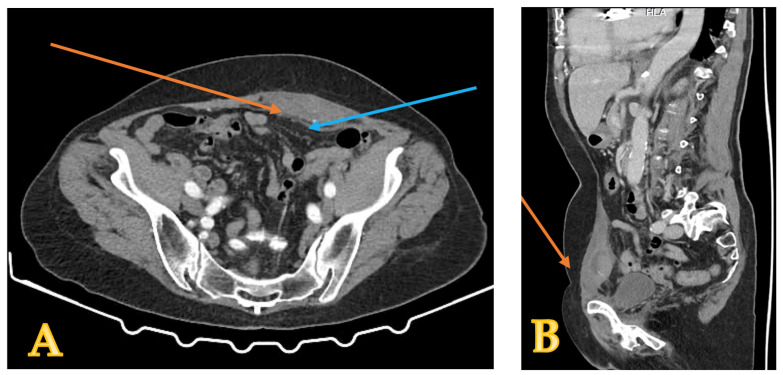
CT scan showing an abdominal mass in the left rectus sheath (orange arrow), near the inferior epigastric artery (blue arrow). The left image (**A**) depicts the axial aspect while the right (**B**) image presents the sagittal aspect of the CT scan.

**Case report number two:** A 60-year-old woman with no history of trauma arrived at the emergency department complaining of sharp pain in her lower abdomen. She had been coughing for the last seven days, and the pain intensified when coughing or sneezing. Medical history revealed usage of anticoagulants for surgical aortic valve replacement; the International Normalized Ratio (INR) was 6.6 on admission, much above the therapeutic accepted limit (for patients not receiving anticoagulant treatment, the INR is usually 1.0; for patients receiving anticoagulant treatment, the therapeutic INR ranges from 2.0 to 3.0 [7]). Clinical examination revealed an abdominal lump in the lower abdomen on the left side; a computed tomography scan characterized this mass as a hematoma in the left rectus sheath (Figure 3).

**Figure 4:**
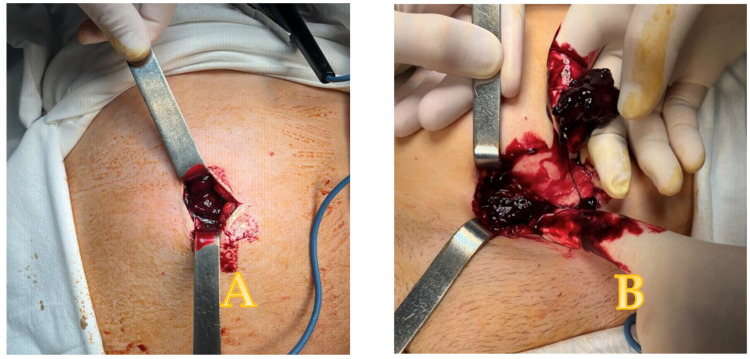

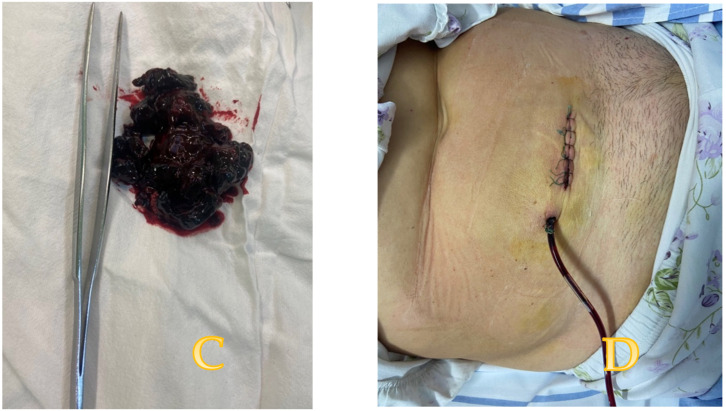
(**A**–**D**) Intraoperative aspect (**A**,**B**), evacuated blood clots (**C**), and postoperative aspect with incision and drain in place (**D**).

The treatment started with correcting the coagulability deficiency: plasma, K vitamin, analgesics, non-steroid anti-inflammatory drugs (NSAIDs), and low-molecular-weight heparin (LMWH)—Clexane 60 mg twice a day. The INR value lowers below 2 to 1,2. Excepting the return of the INR value within normal range, continuing NOM showed no clinical benefit, and the pain did not subside. So, due to the persistence of pain despite actual treatment, a surgical procedure for hemostasis became the main therapeutic option. During surgical exploration, we found and removed multiple clots and uncoagulated blood in the rectus sheath. The remnant cavity was then washed with saline and drained (Figure 4).

The drain remained on site for nine days (until 50 mL). The postoperative evolution was uneventful. The patient underwent careful observation for any possible sign of complications, and frequent ultrasound evaluations confirmed the absence of any residual collection. The patient left the hospital with instructions for additional treatment and follow-up, including the requirement for routine ultrasound examinations to check for a potential hematoma recurrence.

**Figure 5:**
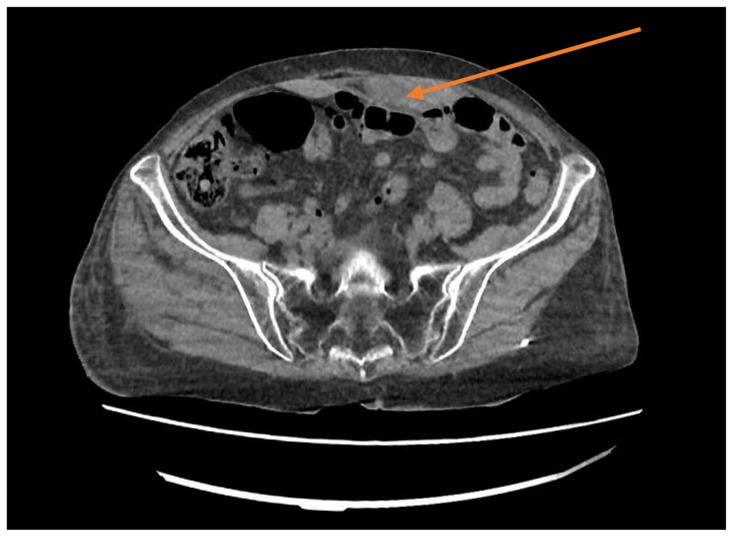
CT scan showing an abdominal mass in the left rectus sheath (orange arrow).

**Case report number three:** A 69-year-old woman with a history of hypertensive heart disease, a prior stroke, and bilateral carotid thrombo-endarterectomy arrived at the emergency department due to pain in the left hemi-abdominopelvic region. Around six hours before her arrival, the patient had identified a left sub-umbilical paramedian tumor following an unsuccessful position change after a prolonged coughing episode. The patient stated that she had developed several widespread ecchymoses since beginning treatment with clopidogrel. Clinically, the patient was hemodynamically and respiratory stable, but she had several ecchymoses on the arm, right shoulder, thigh, and right leg, all at different stages of resolution. The clinical examination upon admission revealed a tumor measuring roughly 10/6 cm, sensitive to touch on the left paramedian area, located in the hypogastrium and extending towards the left iliac fossa, with no visible ecchymoses noted during the inspection. Laboratory evaluations reveal moderate anemia (Hb 8.8 g/dL), but coagulation tests were significantly impaired, showing an INR of 7.5 upon admission. An abdominal CT scan with contrast revealed a newly formed hematoma situated at the level of the left rectus abdominis muscle, measuring 5 × 4 × 9 cm and exhibiting signs of ongoing bleeding (Figure 5).

There are no post-traumatic alterations in the abdominal parenchymal organs, and there are no fluid collections, either intra- or extraperitoneal. Due to the significant coagulation dysfunction, emergency treatment aimed at correcting the coagulopathy first. In addition, the patient received LMWH in conjunction with antibiotics, anti-inflammatory, hemostatic, analgesic, and water electrolysis therapy to restore equilibrium. The patient’s hemodynamic status was stable during this treatment, hemoglobin levels were constant, and abdominal pain symptoms were relieved. Considering the patient’s frailty due to numerous concomitant diseases and the good progress of the treatment started after admission, the medical team decided to refrain from surgery and to continue the initially established treatment under clinical supervision and ultrasound monitoring. Examination of the hematoma was performed initially every 12 h and then daily with ultrasound scans. Ultrasound scans for monitoring the size of the parietal hematoma at the patient’s bedside showed that it was size-stable during the first hours after admission and slowly reduced in size over the following days. Further recovery with conservative treatment allowed the patient to leave the hospital 8 days after admission. At discharge, the patient’s general condition was good, hemodynamics and respiratory function were stable, and there were no signs of peritoneal irritation and parietal bleeding during the healing process. At the follow-up examination one month later, the ultrasound scan showed no accumulation in the rectus sheath.

## 3. Discussion

Cough-induced abdominal wall hematoma, particularly involving the rectus sheath, is a rare and sometimes difficult-to-diagnose condition. Most commonly, the rectus sheath is involved, the anatomical details of the abdominal wall being essential for understanding the mechanism of this type of injury. RSH typically results from the laceration of the rectus muscle or damage to the epigastric arteries during forceful contraction, such as coughing or sneezing. Regarding the anatomy of RSH, there are two main arteries involved. The inferior epigastric artery, a branch of the external iliac artery, penetrates the rectus muscle at the arcuate line and is relatively fixed. Therefore, its branches are more prone to being injured. The other vessel, the superior epigastric artery, is a terminal branch of the internal thoracic artery and has a small caliber; usually, it is implied in minor bleeding and is less common [8]. The pathophysiology involves tearing muscles or rupturing these arteries due to the force generated by a cough. Additionally, the location of the hematoma can vary. However, it is most frequently seen below the arcuate line because the posterior sheath is deficient in this area, making it more vulnerable to injury.

The main circumstances that favor the occurrence of RSH are related to trauma, severe cough, vomiting, anticoagulant use, obesity, coagulopathy, corticosteroid therapy, and SARS-CoV-2 infection, but it also occurs during pregnancy [9,10] and postpartum [11,12]. These factors contribute to the development of RSH in the following ways:

**Hypertension:** Chronic hypertension can weaken arterial walls, particularly smaller vessels like the epigastric arteries, making them more prone to rupture under increased stress, such as violent coughing. Hypertension also complicates clot formation, exacerbating bleeding [13].

**Arteriosclerosis:** Arteriosclerosis leads to the hardening and loss of elasticity in the arteries, predisposing vessels to injury during sudden pressure changes. Atherosclerotic vessels are calcified, and their brittle nature makes them more vulnerable to tears [14].

**Aged population:** Age-related changes in vessel walls, combined with decreased tissue elasticity and impaired healing, increase the risk of RSH. Older adults often have multiple comorbidities, including hypertension and arteriosclerosis, compounding the risk [14].

**Obesity:** The increased intra-abdominal pressure due to excess body weight puts additional strain on the abdominal wall and the rectus sheath in obese individuals. Being overweight can lead to a higher risk of tearing the rectus muscle or injuring the epigastric vessels, particularly during episodes of forceful coughing or physical exertion [8].

**Pregnancy:** The intra-abdominal pressure increases as the uterus enlarges, particularly in the later stages of pregnancy. Additionally, hormonal changes during pregnancy can affect vessel integrity. These factors, combined with any coagulopathies related to pregnancy, make pregnant women more susceptible to RSH [10].

**Corticosteroids:** Long-term corticosteroid treatment can impede angiogenesis and retard wound healing [15,16]; hence, it can influence the occurrence of hematomas. Experiments have demonstrated that corticosteroids can inhibit transforming growth factor-beta and insulin-like growth factor 1 and diminish hydroxyproline, a surrogate marker of collagen formation, leading to decreased elasticity within the arterial bed [17,18].

**SARS-CoV2:** Coagulation dysfunction seemed to be an important issue in patients with COVID-19. The COVID-19 infection leads to severe derangement of hemostasis and prominent alteration of coagulation parameters, promoting a systemic inflammatory response. The severe cases often had prolonged Prothrombin Time (PT), increased D-dimer levels, low fibrinogen, and disseminated intravascular coagulation (DIC) [19].

Secondly, thromboprophylaxis was beneficial in hospitalized patients with COVID-19, but it can lead to a higher-than-usual incidence of RSH in these patients [20,21,22]. Similarly, some authors have reported that surgical management of RSH in COVID-19 patients is superior to interventional radiology [23].

The appearance of a non-traumatic, so-called “spontaneous” hematoma in the rectus abdominis sheath is explained by several mechanisms:

- **Severe cough** generates a brutal contraction (over-contraction) of the abdominal wall muscles and consecutive muscular fibers/epigastric vessel rupture, which further leads to accumulation of blood in the rectus sheath, and pain occurs [23,24,25,26,27,28,29]. A similar mechanism may occur in vomiting, twisting, and sneezing.
- **Anticoagulant medication** is known to play a significant role in developing spontaneous, non-traumatic abdominal wall hematomas, including RSH. By interfering with the body’s natural clotting mechanisms, anticoagulants increase the risk of bleeding, even from minor vessel injury or muscle tears, which otherwise would be self-limited [2,30,31,32]. The mechanism by which anticoagulants contribute to RSH involves the activation of inhibitory pathways in coagulation. When anticoagulant therapy is in place, either prophylactically or therapeutically, it can prevent clot formation even in areas where mild trauma, such as a vigorous cough, leads to vessel injury. The lower coagulability makes anticoagulants the most common iatrogenic cause of such hematomas.
- **Coagulopathies and hematological disorders**, along with diseases affecting the vessel wall, can increase the susceptibility to conditions like RSH, especially in patients with hypertension, arteriosclerosis, advanced age, obesity, and during pregnancy [29].

The interplay of these conditions with coagulopathy or anticoagulation therapy further increases the risk of spontaneous bleeding into the rectus sheath. Recognizing these risk factors is crucial for early diagnosis and effective management of RSH in vulnerable populations. The clinical presentation of RSH typically involves acute abdominal pain, followed by the development of an abdominal wall mass diagnosed by palpation. A decrease in hemoglobin of more than 0.4 g/dL is also a common finding, aiding in diagnosing RSH.

Sometimes, an abdominal wall ecchymosis (Grey Turner’s sign—flank ecchymosis; Cullen—periumbilical area ecchymosis; Fox sign—ecchymosis of the upper thigh with a sharply defined superior border paralleling and inferior to the inguinal ligament; and Bryant sign—blue discoloration of the scrotum) can be found [33]. This sign was observed in our first patient but developed late during the postoperative course, so it cannot be considered as a reliable sign for an early diagnosis. Additional findings include nausea/vomiting and tachycardia. These signs can appear initially or manifest after several days. As Guldner et al. suggest, they can indicate an acute hemorrhage, a subacute process, or a relapse of temporarily stopped bleeding [33,34]. Rarely, peritoneal irritation, hypotension, and fever can develop [2]. It is noticeable that in aged people receiving anticoagulant therapy, spontaneous RSH can present as an acute surgical abdomen, as Salemis et al. state [35]. Ultrasound is the first imaging diagnosis method, but it can sometimes be inconclusive. Hence, it can be used in emergencies and at the bedside to ease differential diagnosis [14,36,37].

The procedure of choice in definitive diagnosis is the CT scan. Its sensitivity and specificity approach 100%. Berna et al. classified RSH into three grades [13]:

- Intramuscular, unilateral, and does not dissect along the fascia planes—mild severity and usually without any hemodynamic disturbance. Commonly do not require hospitalization, but follow-up for eventual recurrence or aggravation is mandatory.
- Intramuscular, more often bilateral, but the blood does not occupy the prevesical space between the transversalis fascia and muscle. These are moderate to severe hematomas, and do require hospitalization, but surgery is not required in most of the cases. Patients who undergo nonoperative management of RSH generally have a favorable evolution but may necessitate a longer hospital stay than operated-on patients.
- Bilateral—these hematomas are generally large and dissect the space between the fascia transversalis and the muscle, occupying the prevesical space and breaking the peritoneum with secondary hemoperitoneum. Such hematomas are almost every time developed in patients undergoing anticoagulant therapy and present with significant hemodynamic disturbance, or even as an acute abdomen [14].

The accuracy of a CT scan in diagnosing RSH is advocated by many authors [38,39]. RSH is a self-limiting condition in some instances, but for most patients, in the absence of treatment, complications can occur. Of these, compartment syndrome and hemodynamic instability are feared complications and can lead to multiple organ failure syndrome (MOFS) and death. The reported mortality in RSH is about 4% in the general population, but it can reach up to 25% in patients with anticoagulant therapy [39,40,41]. Usually, type I and II RSH are well controlled by conservative treatment: analgesia, rest, treatment of the anemia, local compression, ice, alleviating coagulopathy and thrombocytopenia, limited ceasing of the anticoagulants, Greenfield filter (for the IVC), and others [8].

In type III hematomas, or when conservative treatment of the bleeding has failed, angiographic embolization and, or surgical exploration are required [42]. Masuda et al. consider that these are high-risk cases that need prompt treatment [43]. For such cases, besides active bleeding identified by CT angiography, there are multiple risk factors incriminated: volume of the hematoma > 1000 mL, chronic use of corticosteroids, age > 65 years, and transfusion of more than 4 units of red blood cells. Elderly individuals tend to have reduced muscle mass. Therefore, tamponade hemostasis has a debilitated capacity to prevent intramuscular bleeding [43,44].

Elderly patients with comorbidities are usually poor surgical candidates; hence, Tseng et al. suggested that early empiric transcatheter arterial embolization may be appropriate for these cases [45].

Treatment of RSH should be performed in a multidisciplinary setting, with internal medicine, radiology, and surgery included [46].

Transarterial embolization is a fast, safe, and effective procedure for treating RSH hematomas unresponsive to conservative therapy [47]. This procedure is valuable in hemodynamically stable patients and has demonstrated its effectiveness in COVID-19 patients [48,49]. A CT scan determines active bleeding and usually leads to surgical exploration. Surgical treatment can also be opted for in infected hematomas and persistent abdominal pain, and similarly, in cases where angiography and embolization are not available [48].

In the follow-up for patients with RSH, the ultrasound scan proved extremely useful for monitoring gradual resorption.

## 4. Conclusions

Rectus sheath hematoma (RSH) is a rare entity usually associated with trauma; however, in some cases, it can occur after minimal trauma, especially in patients receiving anticoagulant treatment.

The presence of anticoagulants not only predisposes patients to RSH but also makes the management of the hematoma more challenging. Hemostasis may be difficult to achieve, despite replacing anticoagulant oral medication with LWMH. In severe cases, patients may require reversal of anticoagulation, transfusions, or even surgery.

Given the increased use of anticoagulants in clinical practice, vigilance for RSH in patients presenting with abdominal pain and a history of coughing is essential. Typical treatment is a conservative therapy, as this pathologic entity is usually self-limiting. Embolization therapy may be helpful in selected patients, but surgical management is mandatory in hemodynamic instability.

One of our patients had a history of severe comorbidities that made her a poor surgical candidate. In such cases, surgical abstention under close surveillance that allows the decision for surgery at any moment may be a better solution than an early intervention with extremely high risks.

Even though our cases belong to grade II in the Berna classification, in two of them, the failure of the initially conservative management led to a mandatory surgical therapy that finally proved successful.

## Data Availability

The original data presented in this study are available on reasonable request from the corresponding author. The data are not publicly available due to privacy concerns.

## Abbreviations

The following abbreviations are used in this manuscript:

LMWH	Low-Molecular-Weight Heparin
NOM	Nonoperative management
NSAID	Non-Steroidal Anti-Inflammatory drugs
RSH	Rectus Sheath Hematoma
